# Crisis and Emergency Risk Communication and Emotional Appeals in COVID-19 Public Health Messaging: Quantitative Content Analysis

**DOI:** 10.2196/56854

**Published:** 2024-09-17

**Authors:** Shirley S Ho, Agnes S F Chuah, Vanessa S Ho, Sonny Rosenthal, Hye Kyung Kim, Shannon S H Soh

**Affiliations:** 1 Wee Kim Wee School of Communication and Information Nanyang Technological University Singapore Singapore; 2 School of Biological Sciences Nanyang Technological University Singapore Singapore

**Keywords:** COVID-19, crisis and emergency risk communication, CERC, emotional appeal, content analysis, public health, Facebook, social media, Singapore

## Abstract

**Background:**

Although COVID-19 is no longer a global health emergency, it remains pervasive in Singapore, a city-state situated in Southeast Asia, with periodic waves of infection. In addition to disease management, strong communication strategies are critical in the government’s response to the pandemic to keep the public updated and equip them in protecting themselves.

**Objective:**

Grounded in the crisis and emergency risk communication (CERC) framework and emotional appeals, this study aimed to analyze public health communication strategies in Singapore during the COVID-19 pandemic.

**Methods:**

Quantitative content analysis was conducted on 696 Facebook (Meta Platforms Inc) posts and 83 website articles published by Singapore-based public health institutions between January 2020 and September 2022.

**Results:**

The results showed that increasing communication on message themes, such as inquisitive messaging and clarification, can enhance communication strategies. The use of emotional appeals also varies with time and should be carefully used as they are context-specific.

**Conclusions:**

Theoretically, this study contributes to advancements in the CERC framework and concepts of emotional appeals by exploring the applications and changes of CERC message types and emotional appeals at different phases. The findings can provide practical guidance for authorities and communication practitioners in developing effective communication strategies.

## Introduction

### Background

Singapore effectively managed COVID-19, which is evident from the World Health Organization lauding its “all-of-government” approach [1]. This approach [1,2] entails collaboration among different government agencies [2]. While COVID-19 is no longer a global health emergency, Singapore continues to experience periodic infection waves [3]. During the pandemic, the Singaporean government charted its response to COVID-19 in stages, as detailed in a white paper [4]. Avenues for public health communication in Singapore include government websites and Facebook (Meta Platforms Inc) pages. These websites serve as a one-stop communications channel, and Facebook is one of Singapore’s most widely used social-networking platforms [5]. However, studies on the government’s use of Facebook for public health communication during the pandemic are limited.

Singapore’s success in managing the pandemic can be attributed to its small population, concentrated political authority, high political trust [6], state-supported media, and the 2003 SARS outbreak experience [7]. Despite this, Singapore faced criticism for the high number of COVID-19 cases in dormitories of migrant workers, due to the lack of communication [6]. Studies [8-13] have shown that media messages can shape public knowledge, attitudes, and preventive behaviors during pandemics in Singapore. It is worthwhile to study Singapore’s public health communication during COVID-19 as it can highlight areas of improvement and offer insights for other countries in future crisis. This study had 4 objectives. First, it aimed to characterize the themes of public messages during the COVID-19 using the crisis and emergency risk communication (CERC) framework. Second, it aimed to examine how these message themes changed across different pandemic phases. Third, it aimed to identify the types of emotional appeals used. Fourth, it aimed to analyze how emotional appeals changed across the COVID-19 phases.

### CERC Framework

CERC is well-suited for evaluating Singapore’s public communication strategies during the COVID-19 pandemic. This is because CERC evolved in stages and involves both risk and crisis communications. CERC consists of 5 stages: precrisis, initial, maintenance, resolution, and evaluation. Communication during the *precrisis* stage focuses on educating the public about potential adverse events and risks to prepare them for the subsequent stages [14]. During the *initial* stage, communication messages focus on reducing uncertainty, conveying empathy, and imparting the general understanding of the crisis. The *maintenance* stage reiterates misinformation, ongoing risks, and mitigation strategies [15]. The *resolution* stage involves communicating how the emergency was handled, while the *evaluation* stage assesses response effectiveness [15].

The CERC framework assumes that crises develop in a linear way. However, due to the variability of diseases, crises may not follow the sequence of the outlined stages [14]. Although CERC suggests 5 stages, the precrisis stage did not apply to COVID-19 because it was not a known disease. The length of each stage may also vary, as a prolonged crisis state may occur [14]. For example, COVID-19 had a prolonged CERC maintenance stage as the virus mutated several times during the pandemic [16]. This has resulted in repeated tightening and easing of COVID-19 measures in Singapore [4].

### CERC Themes

Drawing on the existing literature [17-19], this study categorized the CERC message themes into 4 categories: *risk and crisis information*, *self-efficacy and sense-making, preparations and uncertainty reduction*, and *advisories and alerts*. *Risk and crisis information* refers to information that educate the public about potential threats [14]. This category consists of a subtheme, *pandemic intelligence.* It refers to messages containing basic information about the pandemic, including case numbers [18], to raise awareness of the current situation.

The category *self-efficacy and sense-making* involves messages that help people to understand the situation and reflect their ability to change their behaviors [18]. This category includes 3 subthemes: *personal preventive measures and mitigation, social and common responsibility,* and *inquisitive messaging*. *Personal preventive measures and mitigation* refers to messages about measures or precautions that can be taken to protect the public from COVID-19. *Social and common responsibility* includes messages on measures or precautions that can be taken at the community level to prevent the spread of COVID-19 or to show care [20]. *Inquisitive messaging* addresses the public’s questions to better understand the situation [18].

The category *preparations and uncertainty reduction* includes messages on how to act appropriately during the pandemic [14]. Drawing reference to Malik et al [18], *preparations and uncertainty* comprises 4 subthemes: *clarification, events, campaigns and activities, showing gratitude,* and *reassurance*. Clarification refers to messages addressing misunderstandings and untrue claims about the pandemic [20]. Events, campaigns, and activities include messages promoting communication campaigns for awareness, relief, or treatment. Showing gratitude refers to expressing appreciation to those involved in managing the virus, such as frontline workers [19]. Reassurance consists of messages that allay the public’s fears*.*

The category *advisories and alerts* refers to messages that provide crucial warnings and specific advice about diseases. There are 2 subthemes: *risk groups* and *general advisories and vigilance*. The subtheme *risk groups* refers to messages targeting susceptible groups such as people with preexisting conditions and older adults who are at greater risk of contracting COVID-19 [20]. Messages on *general advisories and vigilance* include information on what to do in certain situations, such as returning to the workplace.

### COVID-19 Phases and Social Media Use in Singapore

The Singapore government segmented the COVID-19 pandemic into 4 phases: *early days of fog, fighting a pandemic, rocky transition*, and *learning to live with COVID-19* [4], which correspond to the CERC stages (Table 1). However, empirical investigation is needed to examine whether the message themes were conveyed appropriately across these stages, especially on social media. The CERC framework has been used to evaluate public health communications on social media such as Facebook [17,21]. Vijaykumar et al [22] found out that information disseminated by Singapore-based public health institutions on Facebook were similar in content, but differed in focus. The Ministry of Health (MOH) focused on situational updates and the National Environment Agency (NEA) elaborated on preventive measures. However, the study only focused on public communication by these 2 agencies. To gain a broader understanding of crisis communications in Singapore; this study examined public communication by multiple government agencies in Singapore. Hence, we ask the following research questions (RQs):

- RQ1: To what extent are the CERC message themes present in Singapore’s online public health messaging during the COVID-19 pandemic?
- RQ2: How do the CERC message themes change across different phases during the COVID-19 pandemic?

**Table 1:** White paper stages.

CERC^a^ stages	White paper stage	Definition
Initial phase	Early days of fog: January, 2020, to March, 2020	COVID-19 was first presented as a global health crisis as an unknown virus. Singapore detected its first few cases [4]. The Singapore government used the color-coded DORSCON^b^ (MOH^c^ [23]), to reflect the disease situation in Singapore. The DORSCON level was raised from green to yellow, and subsequently to orange. This matches the initial phase of CERC, as the virus had already evolved into a crisis. In this period, uncertainty about the new disease was rife, which is characteristic of the initial phase [15].
Maintenance stage	Fighting a pandemic: April, 2020, to April, 2021	This phase began with Singapore entering a “circuit breaker” phase on April 7, 2020, with increased safe distancing measures to reduce the spread of COVID-19 (MOH [24]), as the situation continued to worsen. Work-from-home was mandated; dining-in was prohibited. Vaccinations were made available first for health care workers and then for older citizens [4]. This corresponds to the maintenance stage proposed by CERC when the COVID-19 situation in Singapore began to stabilize, with Singapore exiting the circuit-breaker stage and gradually relaxing other safe-management restrictions.
Maintenance stage	Rocky transition: May, 2021 to November, 2021	Singapore learns to live with endemic COVID-19. The newly discovered COVID-19 Delta variant caused a large increase in cases, supporting that the “zero COVID” strategy would not be practical in Singapore. In response to the fluctuating COVID-19 cases [4], there were multiple tightening and easing of measures during this period. This also corresponds to the maintenance stage, as outlined by CERC.
Resolution stage	Learning to live with COVID-19: December, 2021, to present	This phase saw further relaxation of safe-management measures. Other measures, such as mask requirements and border measures, were eased. The DORSCON level was stepped down from Orange to Yellow [4]. There was a marked return to normality [20]. Hence, this stage corresponded with the resolution stage proposed in the CERC.

^a^CERC: crisis and emergency risk communication.

^b^DORSCON: Disease Outbreak Response System Condition.

^c^MOH: Ministry of Health.

While CERC is extensively studied, there is limited research linking it with emotional appeals, a gap scholars find crucial to address. Meadows et al [25] argued that investigating the emotional tones of the public during different outbreak phases aids in formulating effective public health messages. This is echoed by Xie et al [26] who found that emotional appeals effectively engaged audiences. In addition to analyzing CERC message themes, this study also aimed to examine the use of emotional appeals in public health communication during COVID-19.

### Emotional Appeals

Emotional appeals can persuade people to perform an intended behavior by evoking specific emotion [27]. They are widely used in health communications [28]; each type elicits varying responses. For example, people are divided on humor appeals; a few think it undermines the seriousness of the subject, while others find it useful [29]. The choice of emotional appeals depends on the context and the target audience [30]. During the COVID-19 pandemic, key emotional appeals included hope, humor, fear, anger, guilt, and nurturance [31,32].

*Hope appeals* emphasize efficacy and can be empowering when paired with actionable advice. During health crises, transparent communication about uncertainties and hopeful messages can enhance support for the measures implemented [32]. World Health Organization recommends using hope appeals to combat pandemic fatigue [33]. Hope appeals are an effective communication strategy across different cultures. In collectivist countries such as Singapore, hope appeals can focus on emerging stronger from COVID-19 as a community. *Humor appeals* use techniques such as clownish humor, irony, and satire [34], aimed at reducing negative emotions and promoting positivity [35]. However, they are also noted for potentially reducing social responsibility [36] and perceived crisis risk [37]. Humor appeals should be used tactfully especially during the critical phrases where increased perceived risk and social responsibility are crucial.

*Fear appeals* are the most widely studied emotional appeals. A message with fear appeals induces fear when a situation is seen as threatening to one’s physical or mental health and is perceived as uncontrollable [38]. It evokes fear about the harm that will befall them if they do not adopt the recommended behavior [39]. The arousal triggered would create a desire to avoid the perceived threat and to adopt the suggested behavior, such as mask wearing and vaccination. Upon encountering the message, the audience would evaluate the severity and susceptibility of the threat, and their ability to overcome the threat, and subsequently taking the recommended action [40].

*Anger appeals* motivate people to carry out actions requiring more effort and commitment [41]. The anger activism model suggests that when coupled with a sense of efficacy, a person made to feel anger would feel motivated to perform a behavior [41]. Anger was one of the least used appeals in organizational YouTube videos during COVID-19 [26]. *Guilt appeals* consist of 2 components—material to evoke guilt and an action to reduce guilt [42]. The material can highlight discrepancies between the audience’s standards and their behavior [43], which could effectively influence health-related attitudes [44]. However, excessive guilt can be counterproductive and less persuasive [45], as shown in the study by Matkovic et al [46], where guilt appeals failed to influence handwashing intention during the pandemic.

*Nurturance appeals* are defined as appeals that evoke a sense of caretaking, which effectively target parents [38]. Nurturance appeals were the most dominant emotional appeal in advertising materials using COVID-19 as a theme [31]. Given the dynamic nature of a crisis, it is important to use suitable emotional appeals at appropriate times and for effective management of the situation. A few studies focused on how emotional appeals were used in the communication messages during the COVID-19 pandemic (eg, a study by Mello et al [47]). Hence, we asked the following RQs:

- RQ3: What are the types of emotional appeals used in Singapore’s online public health messaging during the COVID-19 pandemic?
- RQ4: How does the use of emotional appeals in Singapore’s online public health messaging change across different CERC phases during the COVID-19 pandemic?

## Methods

### Overview

To answer our research questions, we conducted a quantitative content analysis on public Facebook posts and publicly accessible website articles from key Singapore government institutions involved in public health communication during the COVID-19 pandemic. Specifically, we compiled and analyzed content from Gov.sg, representing the Singapore government, as well as institutions, such as the MOH, the Ministry of Sustainability and the Environment, the NEA, and the Health Promotion Board.

### Ethical Considerations

Before commencing data collection for content analysis, we sought approval from the Nanyang Technological University’s Integrity Review Board (IRB-2022-725) in exempt category 4. This category pertained to secondary research using existing or publicly accessible data sets such as those found on social media. The exemption criteria included sources of individually identifiable information that were already in existence or that were publicly available. Obtaining IRB approval ensured that the research adheres to ethical standards, protecting the privacy and rights of individuals whose data were being analyzed. This step was crucial in maintaining the integrity and ethical compliance of the research project.

### Data Collection and Sampling

Upon receiving IRB approval, we used a Python script to crawl Facebook posts containing specified keywords related to COVID-19 from January 1, 2020, to September 30, 2022. Concurrently, we manually compiled relevant website articles from the same timeframe through keyword searches on the institutions’ websites. These keywords included “2019-nCoV,” “SARS-CoV-2,” “Sars-CoV-2,” “Wuhan Coronavirus,” “Wuhan coronavirus,” “wuhan coronavirus,” “Wuhan virus,” “wuhan virus,” “Wuhan Virus,” “Covid-19,” “covid-19,” “novel coronavirus,” “COVID,” “Covid,” and “covid.” Articles and posts that are not related to the public health communication about COVID-19 (such as Facebook posts and website articles that solely focused on situational updates such as the number of cases and clusters, call outs to subscribe for updates, mentions of COVID-19 as a time frame where other activities or programs were the major topics, posts that did not focus on COVID-19, speeches by public figures, and press releases) were excluded.

This initial screening yielded a total of 1114 Facebook posts and 85 relevant website articles. The data were then randomly sampled with a CI level of 99% and a 3% margin of error, resulting in the final 696 Facebook posts and 83 website articles selected for detailed analysis.

### Codebook and Coding Scheme

We developed the codebook on the basis of the CERC message themes adapted from previous literature [18]. These themes encompassed (1) pandemic intelligence, (2) personal preventive measures and mitigation, (3) social and common responsibility, (4) inquisitive messaging, (5) clarification, (6) events, campaigns and activities, (7) request for contributions, (8) showing gratitude, (9) reassurance, (10) risk groups, and (11) general advisories and vigilance. In addition, 6 emotional appeals adapted from previous studies [31,38,48,49] were included in the codebook. These emotional appeals included (1) fear appeals, (2) guilt appeals, (3) anger appeals, (4) hope appeals, (5) humor appeals, and (6) nurturance appeals. Each Facebook post, including all text and visual elements, and everything visible on the webpages were coded as a single unit of analysis.

### Intercoder Reliability

We recruited 3 coders to code the posts and articles. Before conducting actual coding, the coders undertook 2 rounds of training, practice sessions for coding, intercoder reliability, and discussions to refine the codebook. During practice sessions, coders coded the same units of analysis to ensure a common understanding of the codebook. The units of analysis (n=60) for the training and practice sessions included the materials that had not been sampled. After achieving consensus, the coders coded 10% of the data, and intercoder reliability was tested. The process was repeated until we achieved an average Krippendorff α value of 0.78, ranging from 0.70 to 1.00. As it exceeded the 0.70 standard established in the literature [50], demonstrating an acceptable intercoder reliability. Subsequently, the data were split equally and coded by the coders.

### Statistical Analyses

To answer RQ1 and RQ3, a series of descriptive statistics were conducted using SPSS (version 29; IBM Corp). For RQ2 and RQ4, chi-square tests were performed to examine the relationships among CERC themes, emotional appeals, and COVID-19 phases. Notably, 24 website articles lacking publication dates were excluded from the chi-square tests as we could not classify them into any COVID-19 phases.

## Results

Our sample showed that most of the messages about COVID-19 were communicated by Gov.sg (394/779, 50.6%), followed by the MOH (261/779, 33.5%), NEA (90/779, 11.5%), Ministry of Sustainability and Environment (18/779, 2.3%), and Health Promotion Board (16/779, 2.1%; Table 2).

RQ1 asked about the CERC message themes used by the Singaporean government during the COVID-19 pandemic. Our sample (Table 3) showed that most of the messages disseminated during the pandemic were about personal preventive measures and mitigation (522/779, 67%) followed by general advisories and vigilance (445/779, 57.1%); pandemic intelligence (266/779, 34.1%); social and common responsibility (131/779, 16.8%); risk groups (118/779, 15.1%); and event, campaigns, and activities (105/779, 13.5%). A small number of messages showed gratitude (54/779, 6.9%), inquisitive messaging (31/779, 4%), clarification (31/779, 4%), and reassurance (31/779, 4%). Request for contributions (5/779, 0.6%) was least communicated.

**Figure 1:**
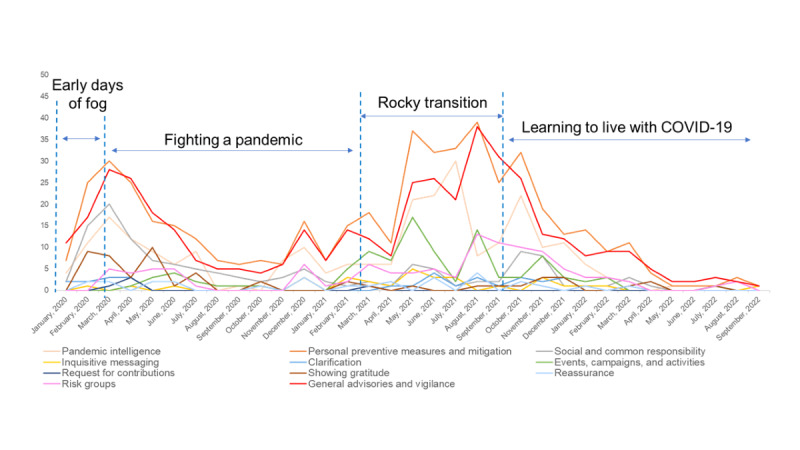
Crisis and emergency risk communication (CERC) stages across phases.

RQ2 asked how the CERC message themes changed across different phases during the COVID-19 pandemic. As shown in Table 4, the communication message themes changed across the COVID-19 phases. Chi-square tests revealed substantial changes in message themes across the phases, including pandemic intelligence (*χ*^2^_3_=18.1; *P*<.001). Specifically, messages on pandemic intelligence were more frequently posted during the maintenance stages—fighting a pandemic and rocky transition—compared with other phases (Figure 1 and Table 4)*.* Similarly, the results showed that message themes such as personal preventive measures and mitigation (*χ*^2^_3_=29.1; *P*<.001); events, campaigns, and activities (*χ*^2^_3_=27.9; *P*<.001); and general advisories and vigilance (*χ*^2^_3_=15.5; *P*<.001) changed significantly across different COVID-19 phases. These message themes were frequently used in Singapore’s online public health messaging during the fighting a pandemic phase and rocky transition phase (ie, maintenance stage).

Chi-square tests showed that message themes on social and common responsibility (*χ*^2^_3_=29.9; *P*<.001) and showing gratitude (*χ*^2^_3_=21.0; *P*<.001) changed across different COVID-19 phases. Messages on social and common responsibility were frequently communicated to the public during the fighting a pandemic period (ie, the maintenance stage), while messages that focused on expressing gratitude were often communicated during the early days of fog (ie, the initial stage) and fighting a pandemic period (ie, the maintenance stage). Message theme on risk groups (*χ*^2^_3_=17.7; *P*<.001) also changed across different COVID-19 phases; messages about risk groups were frequently mentioned during the rocky transition period (ie, the maintenance stage).

RQ3 asked about the types of emotional appeals used in the messages communicated by the Singaporean government to the public during the COVID-19 pandemic. Our data (Table 5) showed that hope (37/97, 38%) and humor (36/97, 37%) appeals were most frequently used in the communication messages during the COVID-19 pandemic, followed by nurturance appeals (17/97, 18%). Anger appeals (4/97, 4%), fear appeals (2/97, 2%), and guilt appeals (1/97, 1%) were used in the messaging strategies with a very low frequency.

RQ4 asked how the use of emotional appeals in messages communicated by the Singaporean government changed across different phases of the COVID-19 pandemic. Chi-square tests (Table 5) showed that emotional appeals—fear, anger, humor, and nurturance appeals—changed across phases. Messages containing fear appeals were only disseminated during the learning to live with COVID-19 period (*χ*^2^_3_=17.4; *P*<.001). Messages containing anger appeals were used during the fighting a pandemic period and learning to live with COVID-19 period (*χ*^2^_3_=8.4; *P*=.04). Humor appeals were used across all the phases of COVID-19 at different levels of frequency (*χ*^2^_3_=8.3; *P*=.04). Messages containing nurturance appeals were also mostly communicated to the public during the learning to live with COVID-19 period (*χ*^2^_3_=49.8; *P*<.001).

**Table 2:** Distribution of posts by health organizations (n=779).

Organization	Posts, n (%)
Gov.sg	394 (50.6)
Ministry of Health	261 (33.5)
Ministry of Sustainability and Environment	18 (2.3)
National Environmental Agency	90 (11.5)
Health Promotion Board	16 (2.1)

**Table 3:** Distribution of CERC^a^ themes.

Themes		Posts (N=779), n (%)
**Risk and crisis information**		
	Pandemic intelligence	266 (34.1)
**Self-efficacy and sense-making**		
	Personal preventive measures and mitigation	522 (67)
	Social and common responsibility	131 (16.8)
	Inquisitive messaging	31 (4)
	Clarification	31 (4)
	Events, campaigns, and activities	105 (13.5)
	Request for contributions	5 (0.6)
	Showing gratitude	54 (6.9)
	Reassurance	31 (4)
**Advisories and alerts**		
	Risk groups	118 (15.1)
	General advisories and vigilance	445 (57.1)

^a^CERC: crisis and emergency risk communication.

**Table 4:** CERC^a^ themes across phases (n=755).

		Early days of fog (January, 2020, to March 2020)		Fighting a pandemic (April, 2020, to April, 2021)		Rocky transition (May, 2021, to November 2021)		Learning to live with COVID-19 (December, 2021, to present)		Total		Chi-square (*df*)^b^	*P* value
		Yes, n (%)	No, n (%)	Yes, n (%)	No, n (%)	Yes, n (%)	No, n (%)	Yes, n (%)	No, n (%)	Yes, n (%)	No, n (%)		
**Risk and crisis information**													
	Pandemic intelligence	32 (28.3)	81 (71.7)	76 (27.3)	202 (72.7)	124 (43.4)	162 (56.6)	27 (34.6)	51 (65.4)	259 (34.3)	496 (65.7)	18.1 (3)	<.001
**Self-efficacy and sense-making**													
	Personal preventive measures and mitigation	62 (54.9)	51 (45.1)	161 (57.9)	117 (42.1)	217 (75.9)	69 (24.1)	58 (74.4)	20 (25.6)	498 (66)	257 (34)	29.1 (3)	<.001
	Social and common responsibility	37 (32.7)	76 (67.3)	53 (19.1)	225 (80.9)	33 (11.5)	253 (88.5)	7 (9)	71 (91)	130 (17.2)	625 (82.78)	29.9 (3)	<.001
	Inquisitive messaging	1 (0.9)	112 (99.1)	11 (4)	267 (96)	15 (5.2)	271 (94.8)	4 (5.1)	74 (94.9)	31 (4.1)	724 (95.9)	4.1 (3)	.25
**Preparations and uncertainty reduction**													
	Clarification	7 (6.2)	106 (93.8)	4 (1.4)	274 (98.6)	15 (5.2)	271 (94.8)	4 (5.1)	74 (94.9)	30 (4)	725 (96)	7.6 (3)	.05
	Events, campaigns, and activities	0 (0)	113 (100)	34 (12.2)	244 (87.8)	56 (19.6)	230 (80.4)	9 (11.5)	69 (88.5)	99 (13.11)	656 (86.9)	27.9 (3)	<.001
	Request for contributions	1 (0.9)	112 (99.1)	4 (1.4)	274 (98.6)	0 (0)	286 (100)	0 (0)	78 (100)	5 (0.7)	750 (99.3)	5.0 (3)	.17
	Showing gratitude	17 (15)	96 (85)	23 (8.3)	255 (91.7)	7 (2.4)	279 (97.6)	7 (9)	71 (91)	54 (7.2)	701 (92.8)	21.0 (3)	<.001
	Reassurance	4 (3.5)	109 (96.5)	12 (4.3)	266 (95.7)	10 (3.5)	276 (96.5)	2 (2.6)	76 (97.4)	28 (3.7)	727 (96.3)	0.6 (3)	.89
**Advisories and alerts**													
	Risk groups	5 (4.4)	108 (95.6)	34 (12.2)	244 (87.8)	55 (19.2)	231 (80.8)	16 (20.5)	62 (79.5)	110 (14.6)	645 (85.4)	17.7 (3)	<.001
	General advisories and vigilance	56 (46.9)	57 (50.4)	140 (50.4)	138 (49.6)	180 (62.9)	106 (37.1)	53 (67.9)	25 (32.1)	429 (56.8)	326 (43.2)	15.4 (3)	<.001

^a^CERC: crisis and emergency risk communication.

^b^In total, 24 website articles were excluded from the chi-square tests as there were no publication dates.

**Table 5:** Emotional appeals across COVID-19 phases (n=97).

	Early days of fog		Fighting a pandemic			Rocky transition			Learning to live with COVID-19			Total			Chi-square (*df*)		*P* value
	Yes, n (%)	No, n (%)	Yes, n (%)	No, n (%)	Yes, n (%)		No, n (%)	Yes, n (%)		No, n (%)	Yes, n (%)		No, n (%)				
Fear appeals	0 (0)	113 (100)	0 (0)	278 (100)	0 (0)		286 (100)	2 (2.6)		76 (97.4)	2 (0.3)		753 (99.7)	17.4 (3)		<.001	
Guilt appeals	0 (0)	113 (100)	1 (0.4)	277 (99.6)	0 (0)		286 (100)	0 (0)		78 (100)	1 (0.1)		754 (99.9)	1.7 (3)		.63	
Anger appeals	0 (0)	113 (100)	2 (0.7)	276 (99.3)	0 (0)		286 (100)	2 (2.6)		76 (97.4)	4 (0.5)		751 (99.5)	8.4 (3)		.04	
Hope appeals	4 (3.5)	109 (96.5)	16 (5.8)	262 (94.2)	11 (3.8)		275 (96.2)	6 (7.7)		72 (92.3)	37 (4.9)		718 (95.1)	2.8 (3)		.41	
Humor appeals	7 (6.2)	106 (93.8)	20 (7.2)	258 (92.8)	7 (2.4)		279 (97.6)	2 (2.6)		76 (97.4)	36 (4.8)		719 (95.2)	8.3 (3)		.04	
Nurturance appeals	0 (0)	113 (100)	1 (0.4)	277 (99.6)	5 (1.7)		281 (98.3)	10 (12.8)		68 (87.2)	16 (2.1)		739 (97.9)	49.8 (3)		<.001	

## Discussion

### Principal Findings

This study examined public health communication strategies in Singapore during the COVID-19 pandemic by applying the CERC framework and emotional appeals. We found that the communication strategies used by the Singaporean public health institutions are aligned with the CERC framework. However, our analysis suggested that CERC message themes, such as inquisitive messaging and clarification, can be conveyed more frequently, particularly at the earliest stage of the crisis. This is in line with CERC recommendations; it also helps in verifying the abundance of information available when there is an infodemic. The COVID-19 phases in Singapore outlined by the government are also aligned with the CERC stages. We found that different emotional appeals were used at various COVID-19 phases in differing situations, which is evident in how nurturance appeals were used to encourage child vaccination, aligned with literature showing that nurturance appeals can effectively target parents. Despite this, certain emotional appeals can be used more frequently at various COVID-19 phases. We observed that Singapore’s communication strategy is aligned with the frameworks of CERC and emotional appeals, with a few areas for improvement as discussed below.

Consistent with the study by Malik et al [18], the findings of this study revealed that Singapore-based public health institutions’ communication themes focused more on personal preventive measures and mitigation as well as general advisories and vigilance. For example, tele-befriending and telecounseling services, such as the Seniors Helpline, were established to help older citizens who face mental distress during the lock-down period. Overall, the Singapore government effectively communicated the message themes recommended by the CERC framework. This is evident from how the framework recommends informing the public about what they can do to protect themselves, the risks of the disease, and the actions that the public health institutions are taking to manage the situation. Meanwhile, the *request for contribution* theme was the one communicated the least, likely due to the Singapore-based public health agencies having sufficient resources to tide over the pandemic. To protect individuals and businesses in the country, the Singapore government had issued multiple budgets and grants since the onset of COVID-19. These monetary payouts include one-off as well as recurring cash grants for individuals whose livelihoods were affected by the pandemic [51]. Assistance was also offered to lower-income households. Examples of this include the COVID-19 Recovery Grant, which ensured that the citizens of Singapore or permanent residents could receive up to SG $700 (US $535) for 3 months if they faced an income loss of at least 50% [51]. The grants were successful in reducing the inequality in Singapore [52].

A shortcoming of the public health institutions’ communication strategies was that messages on clarification was communicated less frequently. This was in line with the existing literature that shows how health care organizations may have insufficient posts addressing misinformation [18,53]. While steps were taken to clarify misinformation and address the public’s questions, there can be more such messaging as COVID-19 was also an infodemic [1,54,55]. Infodemics occur when a large amount of information is rampant, including those that might be inaccurate or confusing [56]. Aligned with Reynolds and Seeger’s [15] argument that communication during the initial phase should aim to reduce uncertainty, the Singapore-based public health institutions can enhance messaging on clarification and inquisitive messaging at the earliest stage of the crisis to prevent outrage and confusion in times of emergency. This is considering the fact that the health institutions would be communicating new information, in the form of pandemic intelligence and general advisories and vigilance, which might lead to increased uncertainty. Separately, the frequency of reassurance messaging can be increased, with the CERC framework encouraging such messaging to be conveyed during the initial and maintenance stages [14]. This can help to assure the public that the health institutions are handling the situation and managing the public’s emotions in times of uncertainty [15].

We found that the communication message themes used by the public health institutions changed across different phases of COVID-19 in Singapore. This finding supported the CERC framework, which suggested that different message themes should be communicated to the public at different stages of a pandemic [26]. For example, we observed that messages on pandemic intelligence were communicated less frequently at the initial stage (ie, early days of fog: January, 2020, to March, 2020) of the COVID-19 pandemic; during this time, there was limited knowledge about the disease. As COVID-19 test kits became available, the Singaporean government could trace the number of cases on a daily basis and better understand the spread of the virus. This enabled them to learn and develop mitigation strategies to control the disease. Hence, there has been an increased focus on communicating messages on pandemic intelligence (eg, messages on the kick-off of COVID-19 vaccination) at the maintenance stage (ie, fighting a pandemic: April, 2020, to April, 2021; rocky transition: May, 2021, to November, 2021) than in other stages. Similarly, as scientists gradually gained more information about the virus, personal preventive measures and mitigation strategies were implemented by the public health institutions and more frequently communicated to the public at the maintenance stage (ie, fighting a pandemic: April, 2020, to April, 2021; rocky transition: May, 2021, to November, 2021). This is in line with CERC’s recommendations to provide more explanations about preventive measures and mitigation strategies during the maintenance stage [20].

Our results showed that positive emotional appeals (eg, hope and humor appeals) were more frequently used in COVID-19 communication strategies. This is in line with the study by Xie et al [26], which found that positive emotions, such as hope, were commonly used in videos on COVID-19. They also posited that positive emotions can be beneficial to public engagement at the start of a pandemic to balance out the public’s negative emotions. Hence, Singapore-based public health institutions may have taken this approach to neutralize the public’s uncertainty. While other studies acknowledge that positive emotional appeals should be leveraged, they also suggested for negative emotional appeals to be used as both types of messages can engage the public in taking up preventive behaviors [53,57]. Positive emotional appeals, such as humor appeals, if overused or applied at inopportune times, can backfire, possibly lowering perceived risk and social responsibility; this may also result in the public not internalizing the intended message or not taking it seriously [58]. In addition, emotional appeals have different effectiveness for different demographics. For example, when compared with younger populations [59], older populations prefer emotional appeals that avoid negative emotional outcomes. Hence, health institutions can consider integrating a mix of emotional appeals for more effective messaging in future public health crises or pandemics.

This study found that the emotional appeals used varied with time, with their use being context-specific, depending on the situation and state of the disease. For example, nurturance appeals were not used at the early stage of the COVID-19 communication but were frequently used during the period of learning to live with COVID-19. This coincided with the first shipment of pediatric doses for the vaccination during the third week of December, 2021 [60], when the government started encouraging parents to bring their children for vaccination. Humor appeals were used with different frequencies across the stages, which could be due to the fluctuating severity of the crisis. Our studies revealed that humor appeals were used in less-pressing messages, such as encouraging the public to take up preventive behaviors, that were more culturally appropriate especially during the stressful pandemic. For example, a sitcom character most Singapore residents are familiar with, Phua Chu Kang, was used in COVID-19 campaign videos that deal with responsible behavior during the pandemic, and later, to boost the local vaccination drive. While humor appeals were used in the communication messages across different stages of COVID-19 pandemic in Singapore, it is recommended that other countries should use the same strategy tactfully. This is because there are many factors, such as relevance and timeliness [61], that could influence the effectiveness of humor appeals. Hence, humor appeals need to be applied in good judgment to avoid unintended outcomes.

By contrast, fear and guilt appeals were less frequently applied in communication messages during the COVID-19 pandemic in Singapore. This demonstrates the Singapore-based health institutions’ careful use of negative emotion appeals in a tense pandemic situation where most people were confined at home during the “circuit breaker” period. Such negative appeals could lead to higher mental stress and compromise social cohesion, if overused. This also explains why fear appeals were used in the later phase of the pandemic (ie, during the “learning to live with COVID-19” phase) when the situation was more relaxed, and most management measures had been eased. Hence, the public health authorities should consider the political and cultural landscape as well as the appropriate junctures when applying emotional appeals in their communication strategies in future.

### Implications and Limitations

Theoretically, this study contributes to the existing literature on both the CERC model and emotional appeals. Apart from exploring how CERC model and emotional appeals were applied in Singapore’s public health communication, this study is one of the few examining the relationship between CERC stages and the use of emotional appeals, especially in the context of COVID-19. This study provides insight on how to use a balanced mix of communication strategies for effective public health communications.

The practical implication of this study is twofold. First, in the local context, the findings of this study could inform Singaporean public health practitioners in developing more comprehensive messages during an emerging health crisis. Understanding how CERC message themes and emotional appeals were used in the public communication strategies during the COVID-19 pandemic could help the relevant authorities identify their strengths and shortcomings. For example, our finding on the lack of clarification messages is a pointer for public communication during the pandemic, especially during the period where misinformation about COVID-19 vaccination for children aged 5 to 11 years in Singapore was widespread [62]. Consequently, the local health authorities can learn from our findings to be better equipped to formulate communication strategies in handling unpredictable and emerging health pandemics in the future.

Second, for other nations, especially those with high population density, the health authorities can emulate Singapore’s communication strategies during the COVID-19 pandemic to structure their communication strategies during the health crisis. In particular, Singapore’s “all-of-government” approach, which involves the collaboration of various government agencies in communicating key messages during crises, is a useful communication strategy. Drawing from Singapore’s approach, other countries could chart their responses in stages during a crisis and formulate timely public health messaging by incorporating CERC message themes together with the appropriate emotional appeal. However, as this study considers CERC message themes and phases and emotional appeals in the context of Singapore, the approach should be adapted with care—given the differences in local governance and culture of each country—because the messages may be received differently, thus affecting communication strategies. The “all-of-government” approach may also need to be tailored as a result.

This study has several limitations. First, this study did not collate Facebook posts and website articles from all the public health institutions and only focused on those that provided pressing information about COVID-19 that are applicable to all members of the public. We did not analyze content from government institutions with more targeted messaging because of the large volume of content for analysis. We also did not analyze other media sources, such as television, radio, newspapers, online news, and other social media content, beyond Facebook because of cross-posting of content. As this study might not provide a complete picture of COVID-19 messaging in Singapore, future research should examine social media posts by various government institutions. Second, website articles without publication dates were excluded from the analyses for RQ1 and RQ2, as we were unable to categorize the data into any of the COVID-19 phases in Singapore. Third, we did not analyze social media responses (ie, likes, shares, and comments) because such information was unavailable for website articles. Future research could examine social media responses for a greater understanding of CERC themes and emotional appeals in the context of COVID-19. Fourth, the findings of this study might not be generalizable to countries that are very different from Singapore because of the country’s specific sociopolitical traits such as its high population density and strong central government. Nonetheless, given its exemplary management of COVID-19, it is worthy of documenting its practice to offer useful insights into future pandemic management. While other countries can learn from Singapore’s approach, there may be a need to tailor the communication strategies according to their characteristics.

Fifth, this study did not specifically focus on messages containing severity and susceptibility because neither theme was encompassed in the CERC model used in this study. Given that severity and susceptibility are important aspects of risk perception, future research should examine these message themes in relation to the CERC model. In addition, this study did not examine the extent to which messaging conveyed acute risks from COVID-19 (eg, hospitalization and death) and chronic risks from COVID-19 (eg, postacute sequelae of COVID-19). Further studies should be conducted to delve into the differences as these may have impacted public willingness to engage in prevention and mitigation behaviors. Finally, while this study examined CERC themes and emotional appeals used across CERC phases, we did not dive into the interaction between CERC themes and emotional appeals. This is a possible area for future studies.

### Conclusion

This study examined public health messaging during COVID-19 pandemic in Singapore. The public health authorities in Singapore have taken a strategic and systematic approach in public health communication coupled with the use of emotional appeal to encourage the public to engage in protective behaviors.

## Abbreviations

CERC: crisis and emergency risk communication
MOH: Ministry of Health
NEA: National Environment Agency

## Data Availability

The data sets generated during and analyzed during this study are available from the corresponding author on reasonable request.
